# Identification of a distinct mutation spectrum in the *SMPD1* gene of Chinese patients with acid sphingomyelinase-deficient Niemann-Pick disease

**DOI:** 10.1186/1750-1172-8-15

**Published:** 2013-01-28

**Authors:** Huiwen Zhang, Yu Wang, Zhuwen Gong, Xiaoyan Li, Wenjuan Qiu, Lianshu Han, Jun Ye, Xuefan Gu

**Affiliations:** 1 Department of Pediatric Endocrinology and Genetic Metabolism, Xinhua Hospital, Shanghai Institute for Pediatric Research, Shanghai Jiao Tong University School of Medicine, Kongjiang Road 1665 #, Shanghai, 200092, China

## Abstract

**Background:**

Clinical observations and molecular analysis of the *SMPD1* gene in Chinese patients with acid sphingomyelinase deficiency Niemann-Pick disease (NPD) are scarce.

**Methods:**

A cohort of 27 Chinese patients diagnosed with acid sphingomyelinase deficiency, within the past five years, were collected and investigated for genotype, phenotype, and their correlations.

**Results:**

The majority of our patients (25/27) were under 18 years of age. From the cohort group, eight (30%) fulfilled characters of type A. Four other patients experienced neurologic involvement after two years of age, these were classified as intermediate type. The remaining fifteen presented without clear neurologic involvement and were regarded as type B. One patient, from the type B group, presented with the unusual symptom of a secondary amenorrhea. Three patients, one from the type B group and two from the intermediate group, presented with pronounced proteinuria, in the late stages of the disease, indicating possible kidney involvement in NPD. Twenty-four *SMPD1* gene mutations had been identified; eighteen of these are novel ones. These included four exonic small deletions/duplications (c.4delC, c.147_150del4, c.842-849dup8, c.1307-1312dup6), one termination mutation (p.Glu248X), and thirteen exonic point mutations (p.Gly336Ser, p.Trp342Cys, p.Leu382Phe, p.Pro429Leu, p.Pro430Ser, p.Trp437Arg, p.Thr451Pro, p.His461Pro, p.Ala484Val, p.Ser486Arg, p.Tyr500His, p.Pro533Leu, p.Val559Leu). Notably, eight mutations had more than one occurrence with c.4delC and p.Glu248X accounting for ~30% of all alleles. Correlation analysis of genotype and phenotype indicated eight mutations, c.842-849dup8, p.Glu248X, p.Arg230Cys, p.Trp437Arg, p.His461Pro, p.Ala484Val p.Ser486Arg, and p.Pro533Leu,to be severe mutations. Five mutations, c.4delC, p.Leu382Phe, p.Pro429Leu, p.Pro430Ser and p.Val559Leu were projected to be mild mutations. Interestingly, three intermediate individuals carried combinations of a mild mutation, c.4delC, on one allele and a severe mutation on the other allele.

**Conclusions:**

The Chinese population may have a comparably high incidence of sphingomyelinase-deficient Niemann-Pick disease type A. This study has identified some novel genotype and phenotype correlations in this rare and devastating disorder.

## Background

Acid sphingomyelinase (ASM) deficient Niemann-Pick disease (NPD) is caused by *SMPD1* gene mutations and subsequent acid sphingomyelinase deficiency. It is a rare autosomal recessive disorder, usually categorized as either neuropathic type A (OMIM: 257200), non-neuropathic type B (OMIM: 607616), or clinically intermediate type [1]. Affected individuals, with the neuropathic type A, present with grossly enlarged spleens and livers, and disease onset at approximately 3 months of age. These individuals also suffer psychomotor development retardation, as evidenced by only achieving milestones of less than a 1-year developmental level, and death occurring at around three years of age. Individuals with type B usually have normal neurological development, and onset of the disease can occur from infancy to late adulthood [2]. In addition to types A and B, an intermediate form exists, this form is differentiated by presence of mental abnormality and onset at 2 to 7 years of age [3].

Occurrence of Niemann-Pick disease type B (NPD-B) is pan-ethnic, while the people of Ashkenazi Jewish descent have a high incidence of NPD-A. The genotype and phenotype correlation for some mutations are consistent, e.g., three common mutations, p.Arg498Leu (p.R496L), p.Leu304Pro (p.L302P), and p.Phe333SerfsX52 (990delC), account for approximately 90% of Niemann-Pick disease type A alleles in the Ashkenazi Jewish population [4]. A common NPD type B mutation, p.Arg610del (R608del), has been reported to be the predominant mutant allele (87%) in Maghreb region of North Africa [5]. A high frequency of this mutation has also been found in Spain (38%) [6], and in a report including patients from European countries, USA and Brazil (25%) [2]. In a worldwide study this mutation accounted for 12% of allelic variation [7]. In another study the p.Gln294Lys (Q292K) mutation was associated with severe and progressive neurological involvement in an intermediate type group [3]. Finally, the p.Trp32X mutation was the most frequent allele identified in an Italian NPD-B cohort study [8].

Currently, the mainstay treatment for Niemann-Pick disease type A/B is symptomatic. Bone marrow transplantation has been performed on a small number of NPD patients and found to be beneficial only to NPD-B individuals [9]. Enzyme replacement therapy in type B has completed phase I clinical trial. A phase II clinical trial should start in the near future. Accurate prediction of disease type from genotype would be critical for optimal treatment choices when a clinical phenotype cannot be determined based on patients’ disease presentation. Data from hundreds of North American, Western European, and Ashkenazi Jewish patients are available. Conversely, data from Chinese patients is rare [1]. In the past 5 years, 27 patients with acid sphingomyelinase deficiency were diagnosed at our center. Here we described their genotypes and compared them with each phenotype to determine any correlations. To date this is the largest mutation profile report on Chinese NPD-A/B patients.

## Methods

### Consent

This study was carried out with approval from the Institutional Review Ethics Board of Shanghai Xinhua Hospital, Shanghai Jiao Tong University School of Medicine. Informed and written consent for the collection of samples was obtained from guardians (for patients under 18 years of age) or adult patients (18 years of age and above).

### Clinical observations

Clinical information involving the symptoms at onset and progression till first examination at the Endocrinology and Genetic Metabolism clinic in Xinhua Hospital was gathered from interviews with parents and from patients’ original medical charts. Special attention was paid to most the commonly occurring symptoms/conditions, such as hepatosplenomegaly, gain/loss in psychomotor developmental milestones, diarrhea, recurrent respiratory inflammation, and blood chemistry panels. Onsite physical examinations consisted of general and neurological examinations, including anthropometric parameters. Retina examinations were not conducted. Patient histories, physical examinations and follow-ups were performed by at least one of the authors.

### Subjects

All subjects were from unrelated families and their parents had no consanguinity. The diagnosis of acid sphingomyelinase deficient Niemann-Pick disease in the majority cases was based on clinical presentations and a low level of ASM activity in peripheral leucocytes. One individual, case 7, was clinically diagnosed by presentation and the detection of Niemann-Pick cells in her bone marrow. The diagnosis was confirmed to be ASM deficiency post mortem; both of her parents were found to carry a “hot” mutation in the *SMPD1* gene.

### ASM activity measurement

Homogenates of leukocytes from patients’ peripheral blood were used to measure ASM activity as previously reported with minor modification [10]. Briefly, leukocytes, isolated from blood cell lysates, were stored at −80°C until analysis. Upon thawing samples were homogenized by sonication. Homogenates were incubated for 17 hours, at 37°C with 1.35 mM 6-hexadecanoylamino-4-methylumbelliferyl-phosphorylcholine (purchased from MOSCERDAM substrates) at pH 5.2. The reaction was stopped using 0.5 M NaHCO_3_, 0.5 M Na_2_CO_3_, and 0.25% Triton X-100 at pH 10.7. A 4- methylumbelliferyl standard was used. The fluorescence signals were read at the excitation wavelength 404 nm and the emission wavelength 460 nm. Protein levels were determined using the Bio-Rad BCA protein assay kit. The normal range of ASM activity is 13.7-86.1 nmol/17 h/mg protein with mean ± SD 47.2 ± 20 nmol/17 h/mg protein.

### Sequencing of genomic DNA

Genomic DNA was extracted from peripheral blood using the RelaxGene blood DNA isolation kit (DNA DP319-01, Tiangen Biotech Co. Ltd., Beijing, China) according to the manufacturer’s protocol. All exons and flanking regions of the *SMPD1* gene were amplified using 4 primer pairs (P1F 5′agaagggtaatcgggtgtcc3′, P1R 5′tagatgccaccctctccatc3′; P2F 5′tggaaatggaggcccaag, P2R 5′ttaggggagccaaatgaaga3′; P3F 5′actgtgagctccttgcaggt3′, P3R 5′tgctcaagggaattttcagc3′; P4F 5′ggggaggctcctcactagaa3′, P4R agctccaggaaaggagaagg3′) and sequenced bi-directionally. The obtained sequences were blasted against the *SMPD1* reference gene (NM_000543) to identify mutations and/or variations. In most cases, patients’ parental DNA samples were analyzed to establish the variation origin.

### RNA expression of *SMPD1* gene

To study the pathogenic effects of a variation in *SMPD1* intron 5, from patient number 8, IVS5+5G>C, RNA was extracted from peripheral blood stored in a PAXgene™ blood RNA tube (Qiagen) as previously reported [11]. RNA was reverse transcribed with PrimeScript ^R^RTase (TaKaRa, Japan) and amplified with two primer pairs (P5F 5′cgtcacagcacttgtgagga3′, P5R 5′ccaggattaaggccgatgta3′; P6F 5′atcggccttaatcctggttac3′, P6R 5′ ggctttttcaccctttcctac3′) with the products sequenced bi-directionally.

## Results

According to the NPD-A/B classification criteria [3], 8 individuals were assessed as type A and 4 as suffering from the intermediate form. The remaining 15 individuals, including 5 patients under 2 years of age, free of neurologic impairment, were classified as type B (Table 1). The possibility does exist that a few type B patients may devolve into the intermediate type. Type A patients accounted for 30% of the study group. In accordance with previous findings, hepatosplenomegaly was detected in all patients. Mildly elevated levels of hepatic transaminases and triglycerides, along with chronic diarrhea, were also common clinical findings. Two intermediate patients (number 9 and 10) and one type B patient (number 25) were in critical condition, presenting with a pot belly, ascites, pitting edema on lower extremities, hypoproteinemia, and proteinuria. These symptoms indicate possible kidney involvement associated with late stages of the disease.

**Table 1:** Clinical and molecular data of 27 Chinese patients with acid sphingomyelinase-deficient NPD

**P**	**NPD type**	**Gender**	**Age at diagnosis**	**Age of onset**	**Phenotype and notes**	**Genotype**	**Amino acid changes**	**Intragenic position**	**ASM activity**
1	A	male	1y	1 m	HP, PR, diarrhea, RG, death at 3 years 5 months	[c.842-849dup8] + [c.742G>T]	p.His284SerfsX17 / p.Glu248X	E2 / E2	0
2	A	female	2y	3 m	HP, PR, RG, PF	[c.842-849dup8] + [c.1451C>T]	p.His284SerfsX17 / p.Ala484Val	E2 / E5	0
3	A	female	11 m	5 m	HP, PG, RG, anemia, leucocytopenia, RR, died at age 1	[c.842-849dup8] + [c.742G>T]	p.His284SerfsX17 / p.Glu248X	E2 / E2	2.3
4	A	male	1y4m	5 m	HP, hypotonia,diarrhea, elevated TG, death at 20 m	[c.1309T>C] + [c.1598C>T; c.1621G>A]	p.Trp437Arg / (p.Pro533Leu; p.Ala541Thr)	E4 / E6	4.9
5	A	male	6 m	3 m	HP, hypotonia, PF	[c.842-849dup8] +[c.842-849dup8]	p.His284SerfsX17 / p.His284SerfsX17	E2 / E2	1.7
6	A	female	3 m	after birth	HP, jaundice, hypotonia,PF	[c.842-849dup8] + [c.742G>T; c.1445T>C]	p.His284SerfsX17 / (p.Glu248X; p.Phe482Ser)	E2 / (E2;E5)	0.6
7	A	female	2y	3 m	HP, hypotonia, RR, died at 1y	[c.842-849dup8] +[c.1458T>G]	p.His284SerfsX17 / p.Ser486Arg	E2 / E5	unknown
8	A	male	1y4m	6 m	diarrhea, HP, hypotonia, PR	[c.1382A>C] +[IVS5+5G>C]	p.His461Pro	E5	3.5
9	intermediate	male	7y	7 m	HP, RG, SS, MR at age 5, ascites, hypoproteinemia, died at age 9 y	[c.4delC] + [c.688C>T]	p.Arg3AlafsX76 / p.Arg230Cys	E1 / E2	3.6
10	intermediate	male	5y	2y	HP, SS, RG, anemia, leucocytopenia, ascites, hypoproteinemia,MR at age 4, died at age 6 y, PF	[c.4delC] + [c.742G>T]	p.Arg3AlafsX76 / p.Glu248X	E1 / E2	3.4
11	intermediate	female	8y	8y	HP, moderate MR	c.1458T>G	p.Ser486Arg	E5	1.2
12	intermediate	male	2y	6 m	HP, RG	[c.4delC] +[c.1458T>G]	p.Arg3AlafsX76 / p.Ser486Arg	E1 / E5	3.8
13	B	male	6y4m	1y	HP, SS, RG, anemia	[c.688C>T] + [c.1288C>T]	p.Arg230Cys / p.Pro430Ser	E2 / E4	0
14	B	male	1y10m	1y	HP, RG	[c.1026G>T] +[1492C>T]	p.Trp342Cys / p.Arg498Cys	E2 / E5	0
15	B	female	1y8m	after birth	HP, PF	[c.1095-1096insG] +[c.748A>C; c.1411G>A]	p.Phe368ValfsX22 / (p.Ser250Arg; p.Glu471Lys)	E3 /(E2;E5)	5.9
16	B	female	22y	22y	amenorrhea, pancytopenia, HP	[c.1144C>T] +[c.1565A>G]	p.Leu382Phe / p.Asn522Ser	E3 / E6	3.1
17	B	male	1y3m	10 m	HP	[c.4delC]+[c.4delC]	p.Arg3AlafsX76 /p.Arg3AlafsX76	E1 / E1	3.6
18	B	male	13y	2y	HP	[c.1458T>G] +[c.1675G>T]	p.Ser486Arg/ p.Val559Leu	E5 / E6	5.6
19	B	female	46y	unknown	HP, splenectomy at age 46 years,PF	[c.1565A>G] +[c.1565A>G]	p.Asn522Ser / p.Asn522Ser	E6 / E6	7.3
20	B	female	2y	1y	HP, RG	[c.759C>A] + [c. 1351 A >C]	p.Glu253Asp/ p.Thr451Pro	E2 /E5	2.75
21	B	female	3y	1y	HP, microproteinuria	undetected			2.4
22	B	female	3y7m	after birth	diarrhea, HP, PF	[c.4delC]+[c.4delC]	p.Arg3AlafsX76 / p.Arg3AlafsX76	E1 / E1	4.8
23	B	female	1y8m	after birth	HP	[c.147-150del4] +[c.1144C>T]	p.Ser50ThrfsX26 / p.Leu382Phe	E1 / E3	2
24	B	male	7y6m	7y	HP, RG, SS	[c.4delC]+[c.4delC]	p.Arg3AlafsX76 / p.Arg3AlafsX76	E1 / E1	1.4
25	B	female	12y	10 m	HP, proteinuria, ascites, RG, splenectomy at age 9, died at age 13	c.1006G>A	p.Gly336Ser	E2	4.9
26	B	female	2y4m	2y	HP, anemia	[c.1286C>T] +[c.1451C>T]	p.Pro429Leu / p.Ala484Val	E4 / E5	3.1
27	B	female	1y	1y	HP, anemia	[c.1307-1312dup6] + [c.1497-1498GT>AC]	p.436-437dup2 / p.Tyr500His	E4 / E6	3

Note: HP indicated hepatosplenomegaly, PR indicated psychomotor regression, RG indicated mildly raised GPT and GST, PF indicated positive family history, RR indicated recurrent respiratory tract infection, SS indicated short stature, MR indicated mental regression. P indicated patient number, and E indicated exon. The mean ± SD of ASM activity was 47.2 ± 20 nmol/17 h/mg protein (range: 13.7 to 86.1).

In this study, biallelic mutations were detected in twenty-three patients and only one mutant allele could be identified in 3 patients (number 8, 11, 25) (Table 1). No mutation in the *SMPD1* gene was detected in one patient (number 21), although twice assay of ASM activity from different preparation of her peripheral leucocytes had been carried out to confirm its deficiency. Several mutations, including small exon deletions (c.4delC, c.147-150del4), duplications (c.1307-1312dup6, c.842-849dup8), small insertion (c.1095_1096insG), and termination mutation (p.Glu248X), resulting in premature stop codon and rendering the enzyme noncatalytic, were considered to be pathologic. Novel and recurrent point variations, such as p.Leu382Phe, p.Ala484Val, and p.Ser486Arg were also regarded as pathologic. For those novel and private variations, their pathogenicities were assessed using PolyPhen-2 (http://genetics.bwh.harvard.edu/pph2/index.shtml). Nine of them, p.Gly336Ser, p.Trp342Cys, p.Pro429Leu, p.Pro430Ser p.Trp437Arg, p.Thr451Pro, p.His461Val, p.Tyr500His, and p.Pro533Leu, were predicted to have a maximum damaging effect score and presumed to be pathological. The variation p.Val559Leu was predicted to be benign, with a score of 0.22. Considering its presence in a type B patient (number 18) with a severe mutation, p.Ser486Arg (presented below), it was regarded as a mutation with mild effect.

**Figure 1:**
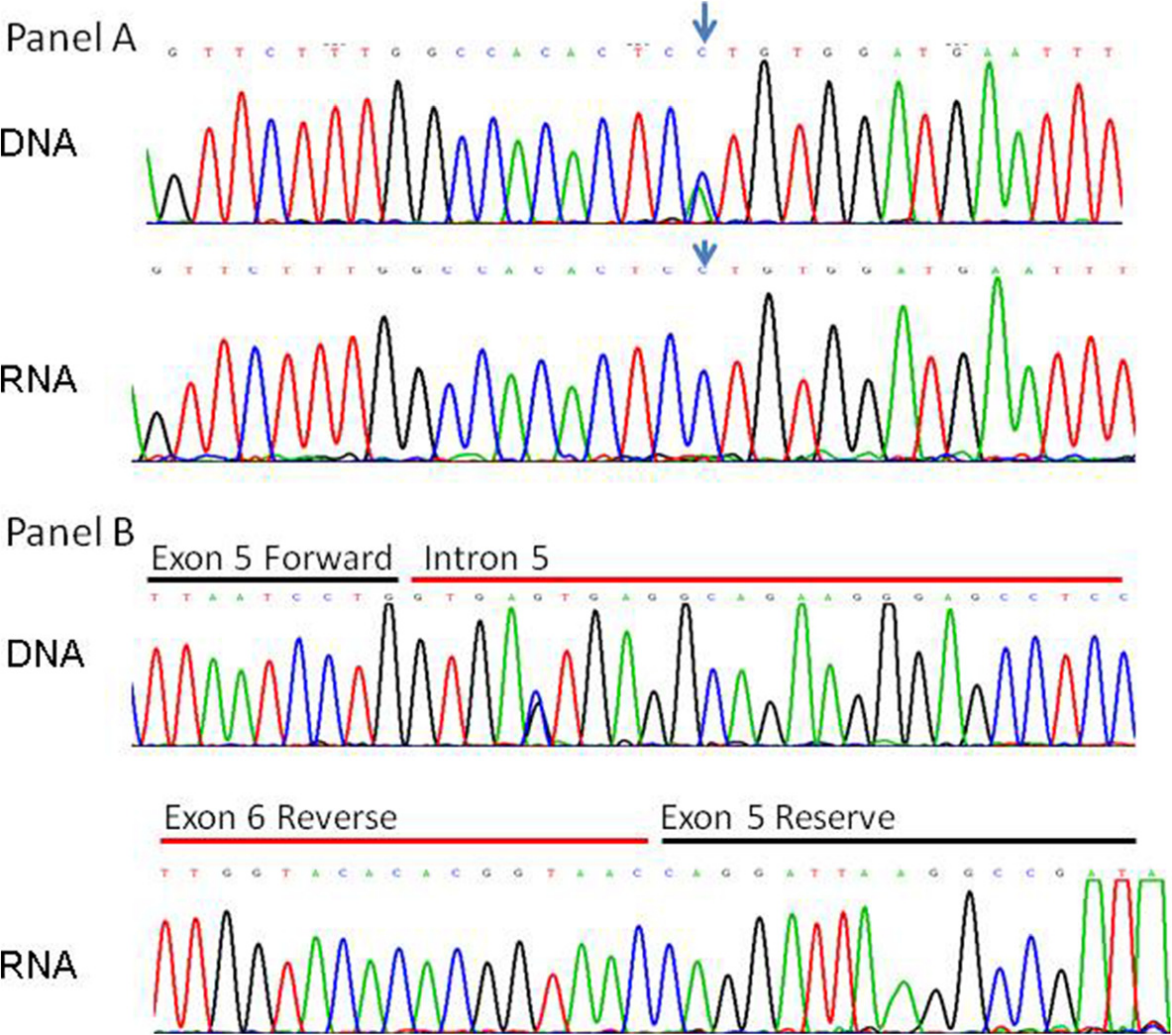
**Failed amplification of paternal allele of the SMPD1 gene in patient number 8.** The panel **A** showed patient 8 carried a heterozygous mutation, c.1382A>C, derived from her mother, as observed on a DNA level. However, at the RNA level the expression was in an apparently homozygous state. The panel **B** showed an intron variation IVS5+5G>C at the DNA level derived from this patient’s father. At the RNA level exon 5 and 6 remained unbroken, indicating lack of amplification of paternal allele.

**Figure 2:**
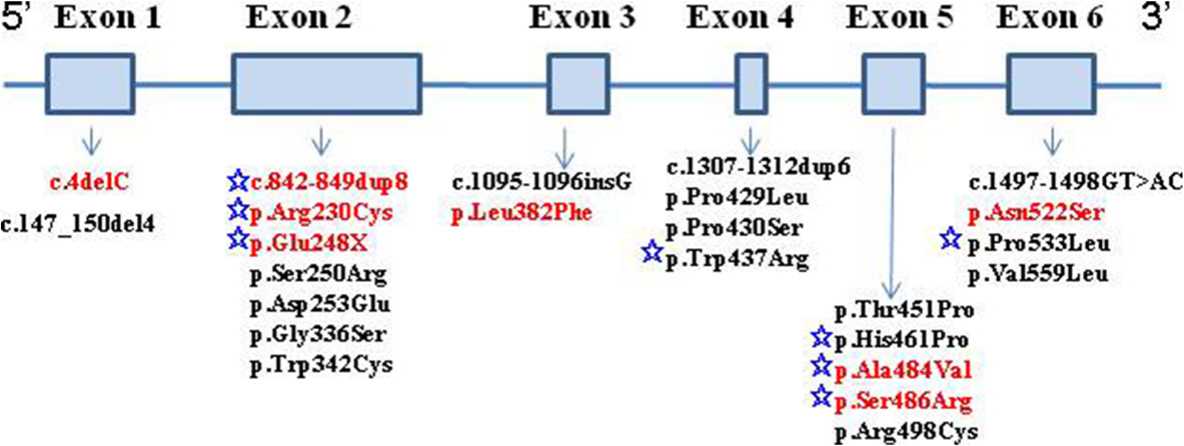
**Distribution of *SMPD1 *gene mutations from Chinese NPD-A/B patients.** A total of 24 different mutations had been identified in this Chinese patient cohort. The eight recurrent mutations are in red, and the presumed severe mutations are marked by blue pentagram.

An intron variation, IVS5+5G>C, derived from a paternal allele was identified in patient number 8. To investigate its pathological influence in this patient, RNA was extracted and analyzed for *SMPD1* gene expression. The result apparently showed patient 8 exclusively expressed his maternal allele carrying a p.His461Val mutation (Figure 1, Panel A), and IVS5+5G>C variation did not have any observable impact on RNA splicing (Figure 1, Panel B). In this patient, the *SMPD1* gene may be paternally imprinted and the maternal allele preferentially expressed, as reported previously [12]. Our inability to detect the mutant IVS5+5G>C allele product could also be due to a premature stop codon and nonsense-mediated mRNA decay. Further functional test, such as a mini-gene system, need to be done to determine the pathogenicity of IVS5+5G>C mutation. In total, at least 24 different mutations had been found (Figure 2).

Three patients (number 15, 6, and 4) carried two sequence variations on a single *SMPD1*allele. In patient 15, both p.Ser250Arg and p.Glu471Lys mutations were of maternal inheritance. Previously, the p.Ser250Arg mutation has been reported in combination with a premature stop codon, in a Dutch patient with a mild form of type A NPD [10]. In this study, we were unable to determine the pathogenicity of p.Glu471Leu. Similarly, in patient 6, a termination, p.Glu248X, and an exon point mutation, p.Phe482Ser, were identified on the same allele. Another variation at the same codon, p.Phe482Leu, had been documented in a type B patient [7]. The nature of p.Phe482Ser was unclear here. Another variation, p.Ala541Thr, was identified on the same allele as p.Pro533Leu, in patient 4. To fully understand the complete effects of these three variations, expression studies need to be conducted as previously reported [10].

It is very interesting that with the exception of five exon point mutations, c.688C>T (p.Arg230Cys) [7], c.748A>T(p.Ser250Arg) [13], c.759C>A (p.Asp253Glu) [14], c.1565A>G (p.Asn522Ser) [10], c.1492C>T (p.Arg498Cys) [15], and a small insertion, c.1095_1096insG [7], the majority of mutations, including c.4delC, c.147_150del4, c.1307-1312dup6, c.842-849dup8, p.Glu248X, p.Gly336Ser, p.Trp342Cys, p.Leu382Phe, p.Pro429Leu, p.Pro430Ser, p.Trp437Arg, p.Thr451Pro, p.His461Pro, p.Ala484Val, p.Ser486Arg, p.Pro533Leu, p.Val559Leu and p.Tyr500His, were novel according the *SMPD1* gene mutation database (http://www.hgmd.cf.ac.uk). These newly detected mutations have been deposited in the public databases dbSNP, with the batch accession number 1057561.

Among all mutations, 8 of them, c.4delC, c.842-849dup8, p.Glu248X, p.Ser486Arg, p.Asn522Ser, p.Leu382Phe, p.Arg230Cys, and p.Ala484Val, occurred more than once and accounted for 61.1% of all disease alleles (Table 2). The two most frequent mutations were c.4delC and c.842-849dup8 and consisted of 29.6% of the total alleles.

**Table 2:** Genotype/phenotype correlation for 8 recurring mutation in Chinese patients

**Mutation**	**Exon**	**Number of Alleles**	**Phenotype**
c.4delC	1	9	3 type B patients in homozygosity; 3 intermediate patients in heterozygosity
c.842-849dup8	2	7	1 type A patient in homozygosity; 5 type A patients in heterozygosity
c.742G>T (p.Glu248X)	2	4	3 type A patients in heterozygosity; 1 intermediate patient in heterozygosity
c.1458T>G (p.Ser486Arg)	5	4	2 type A patients in heterozygosity;1 intermediate patient in heterozygosity; 1 type B patient in heterozygosity
c.1565A>G (p.Asn522Ser)	6	3	1 type B patient in homozygosity;1 type B patitent in heterozygosity
c.1144C>T (p.Leu382Phe)	3	2	2 type B patients in heterozygosity
c.688T>C (p.Arg230Cys)	2	2	1 type B patients in heterozygosity, 1 intermediate patient
c.1451C>T (p.Ala484Val)	5	2	1 type A patient in heterozygosity; 1 type B patient in heterozygosity

Two recurrent mutations, c.842-849dup8 and p.Glu248X, are presumed to cause premature stop codon and are considered severe mutations. In accordance with a severe genotype, 3 individuals (number 1, 3, and 6) carrying a combination of mutations with c.842-849dup8 on one allele and p.Glu248X on the other, had typical type A presentation. Since both severe alleles are required to result in NPD-A and at least one severe allele is required to intermediate type NPD, p.Trp437Arg (in number 4), p.Ala484Val (in number 2), p.Arg230Cys (in number 9), p.His461Pro (in number 8), p.Ser486Arg (in patient 7 and 12), and p.Pro533Leu (in number 4) were estimated to be severe mutations. In consideration that at least one mild mutation is necessary to result in NPD-B, p.Pro430Ser (in number 13), p.Val559Leu (in number 18), p.Leu382Phe (in number 23), p.Pro429Leu (in number 26) were estimated to be mild mutations.

## Discussion

In the largest NPD database, at Mount Sinai School of Medicine, ~20% presented with type A, of these 66% were Ashkenazi Jewish [1]. Although NPD-A has a lower incidence in Chinese NPD patients than in Ashkenazi Jewish, it is still of notable occurrence. In this study, there were only two patients (number 16 and 19) of adult age, the rest ranged in age from 3 months to 12 years, with a mean age of 5.6 years. Therefore, these finding could be biased with respect to patients’ age.

Beside the classical presentation discussed earlier [2,16], growth restriction has been described in patients with NPD-B [17]. In our observations, growth restrictions were more apparent in patients 5 years of age and above. For example, patient 9, 10, 13 and 24 had Z scores for height lower than −2, meeting the criteria of short stature. Conversely, two adult patients (patient 16 and 19), with the p.Asn522Ser mutation, had normal height. Patient 16, an adult, sought medical treatment for secondary amenorrhea initially and was, only then, diagnosed with NPD-B. Amenorrhea is rarely described comorbidity with NPD-B [2]. From this data, we propose the menstrual cycles of adult female NPD patients should be observed.

Although NPD-B is a multi-system disease, kidney involvement is rarely described. Imaging studies found that the kidneys were enlarged by Niemann-Pick cells infiltration [18]. In the end stages of an intermediate NPD patient, kidney biopsies found foamy podocytes, vacuolated tubular epithelial cells, and accumulation of foam cells in the interstitium [19]. In this study, B ultrasonography identified abnormal signals in bilateral adrenal pelvis and bilateral large kidney size in one patient (number 13). Three other patients (number 9, 10, and 25), in the late stages of the disease, presented with proteinuria, hypoproteinemia, and ascites. In addition, patient 21 had microproteinuria. All these observations substantiate kidney involvement in NPD.

In this study we found Chinese patients to be free of “hot” mutations identified in other ethnicities, such as p.Arg498Leu [20], p.Leu304Pro [21], p.Phe333SerfsX52 [22], p.Arg610del [23], p.Trp393Gly [24], and p.Q294K [14]. Here, the recurrent mutations were novel, e.g. c.4delC, c.842-849dup8, p.Glu248X, p.Leu382Phe, p.Ser486Arg, p.Ala484Val. As a group, these mutations accounted for 51.9% of all alleles.

With regard to phenotype/genotype correlations, c.842-849dup8 was homozygous in one NPD-A patient (number 5), heterozygous in five other NPD-A patients (number 1, 2, 3, 6, and 7), and not present in any NPD-B patients, thus c.842-849dup8 is considered a severe mutation. Another mutation, c.4delC, was found to be homozygous in 3 type B patients (17, 22, and 24) and heterozygous in 3 intermediate patients (9, 10, and 12). The oldest homozygous c.4delC patient was 7 years of age and without neurologic involvement (patient 24), which indicated c.4delC is a non-neurotoxic mutation resulting in comparatively low severity of NPD. Interestingly, when considering the position of this mutation, c.4delC was expected to produce a complete enzyme deficiency. However, we observed it was associated with a mild form of the disease. It has been previously reported that a second methionine (Met33) can rescue mutations involving the traditional start methionine [8], this may be occurring in the c.4delC mutation as well.

The c.1565A>G (p.Asn522Ser) mutation resulted in ~10% of wild type activity and has been described previously in a NPD-B patient [10]. Both patients with homoallelic (number 19) or heterallelic (number 16) p.Asn522Ser were free of neurologic involvement. Additionally, the disease onset occurred in their adulthood and the patients had normal statures, supporting the presumed mild nature of the mutation.

Previous finding indicate that patients with the intermediate phenotype carried at least one severe mutation [8]. Here, three intermediate patients (number 10, 11, 12) had one severe mutation, p.Glu248X and p.Ser486Arg (twice occurring) respectively. Besides, three of four intermediate patients had the common c.4delC (number 9, 10, 12), which was assumed to be a mild mutation. On this basis, p.Arg230Cys was presumed to be a severe mutation considering its presence with c.4delC in patient 9. The combination of a severe mutation on one allele and a mild mutation on the other allele indicates their intermediate status both in clinical phenotype and genotype.

In conclusion, a high portion of Chinese NPD patients have been diagnosed with type A. The *SMPD1* gene mutation profiles in Chinese patients are markedly different from reported data of other ethnic groups. Two novel mutations, c.4delC and c.842-849dup8 had significantly high incidences in this cohort of patients. Our data provides novel genotype and phenotype correlation of ASM deficiency and has important implications in genetic counseling and in decisions regarding therapy for care givers. In the future it may aid in large scale carrier screening. One of the shortcomings of this study is that all data comes from one hospital. For this rare disease, data accumulated from multiple centers of China would strengthen these observations.

## Competing interests

The authors declare that they have no competing interest.

## Authors’ contributions

ZH and GX conceived of the study. ZH participated in the sequence alignment and drafted the manuscript. WY and GZ carried out the molecular genetic studies. ZH, YJ, QW, HL, GX participated patient history and examination. All authors read and approved the final manuscript.
